# Hemodynamic effects of enhanced external counterpulsation on cerebral arteries: a multiscale study

**DOI:** 10.1186/s12938-019-0710-x

**Published:** 2019-08-28

**Authors:** Bao Li, Wenxin Wang, Boyan Mao, Yahui Zhang, Sihan Chen, Haisheng Yang, Haijun Niu, Jianhang Du, Xiaoling Li, Youjun Liu

**Affiliations:** 1 Department of Biomedical Engineering, College of Life Science and Bioengineering, Beijing University of Technology, No. 100 Pingleyuan, Chaoyang District, Beijing, 100124 China; 2 Sino-Dutch Biomedical and Information Engineering School, Northeastern University, Shenyang, 110004 China; 3 School of Biological Science and Medical Engineering, Beihang University, Beijing, 100083 China; 4 The Eighth Affiliated Hospital, Sun Yat-sen University, Shenzhen, 518033 China

**Keywords:** Enhanced external counterpulsation, Cerebral artery, Geometric multiscale model, Mean arterial pressure, Cerebral blood flow, Wall shear stress

## Abstract

**Background:**

Enhanced external counterpulsation (EECP) is an effective method for treating patients with cerebral ischemic stroke, while hemodynamics is the major contributing factor in the treatment of EECP. Different counterpulsation modes have the potential to lead to different acute and long-term hemodynamic changes, resulting in different treatment effects. However, various questions about appropriate counterpulsation modes for optimizing hemodynamic effects remain unanswered in clinical treatment.

**Methods:**

A zero-dimensional/three-dimensional (0D/3D) geometric multiscale model of the cerebral artery was established to obtain acute hemodynamic indicators, including mean arterial pressure (MAP) and cerebral blood flow (CBF), as well as localized hemodynamic details for the cerebral artery, which includes wall shear stress (WSS) and oscillatory shear index (OSI). Counterpulsation was achieved by applying pressure on calf, thigh and buttock modules in the 0D model. Different counterpulsation modes including various pressure amplitudes and pressurization durations were applied to investigate hemodynamic responses, which impact acute and long-term treatment effects. Both vascular collapse and cerebral autoregulation were considered during counterpulsation.

**Results:**

Variations of pressure amplitude and pressurization duration have different impacts on hemodynamic effects during EECP treatment. There were small differences in the hemodynamics when similar or different pressure amplitudes were applied to calves, thighs and buttocks. When increasing pressure amplitude was applied to the three body parts, MAP and CBF improved slightly. When pressure amplitude exceeded 200 mmHg, hemodynamic indicators almost never changed, demonstrating consistency with clinical data. However, hemodynamic indicators improved significantly with increasing pressurization duration. For pressurization durations of 0.5, 0.6 and 0.7 s, percentage increases for MAP during counterpulsation were 1.5%, 23.5% and 39.0%, for CBF were 1.2%, 23.4% and 41.6% and for time-averaged WSS were 0.2%, 43.5% and 85.0%, respectively.

**Conclusions:**

When EECP was applied to patients with cerebral ischemic stroke, pressure amplitude applied to the three parts may remain the same. Patients may not gain much more benefit from EECP treatment by excessively increasing pressure amplitude above 200 mmHg. However, during clinical procedures, pressurization duration could be increased to 0.7 s during the cardiac circle to optimize the hemodynamics for possible superior treatment outcomes.

## Background

Enhanced external counterpulsation (EECP) is a noninvasive clinical method which is recommended by the US Food and Drug Administration (FDA) for treatment of cardio-cerebrovascular disease [1]. EECP uses cuffs to mechanically compress the human lower body and increase diastolic blood pressure (DBP) while decreasing compression at the onset of systole and decreasing vascular resistance to reduce the intra-aortic systolic blood pressure (SBP) [2]. By improving blood circulation, EECP assists cardiac function while increasing blood perfusion in the heart, and brain, as well as the kidneys and other organs [3]. This is a common method for the treatment of cerebral ischemic stroke which is globally applied [4–7].

The basic principle of EECP treatment is to significantly increase DBP and form a double-pulse blood perfusion mode for cerebral blood vessels, thus improving cerebral blood flow (CBF). EECP can effectively increase blood perfusion in the brains of patients with ischemic stroke while alleviating ischemia symptoms, which are the acute hemodynamic effects of treatment, in real time. In addition, by accelerating blood flow, EECP significantly improves wall shear stress (WSS) in cerebral arteries. For stenotic cerebral arteries, vascular endothelial cells (VECs) of stenosis are constantly exposed to a high WSS environment throughout the long-term application of EECP, effectively inhibiting atherosclerosis development and promoting the benign remodeling of blood vessels [8]. While the long-term effects of vascular remodeling are complex and do not depend on any single factor, WSS is a clinically recognized indicator which significantly impacts the remodeling and inhibits the development of atherosclerosis. Research has shown that high WSS can promote growth in collateral vessels which have stopped growing, thus significantly increasing numbers of new microvessels in the stenotic region [9]. Therefore, when vascular stenosis occurs, local high WSS in the plaque promotes the formation of microcirculatory vessels, leading to blood perfusion in the ischemic region through the separation of blood flow.

However, further research [10–14] demonstrates that low WSS (< 1 Pa) may promote the development of plaque, while excessive WSS (> 7 Pa) can make plaque unstable and vulnerable to rupture. Moderately high WSS (1 < WSS < 7 Pa) may affect vascular endothelial cell gene expression, promote cell growth and energy metabolism, decrease intracellular lipid deposition, as well as decrease cell adhesion and immune inflammatory response. WSS has the function of protecting the endothelial layer and promoting repair of damaged blood vessels. As a result, moderately high WSS is beneficial for the benign remodeling of stenotic vessels and inhibiting the development of atherosclerosis. As well as WSS, high oscillatory shear index (OSI) is also a predictor of atherosclerosis and vulnerable plaque [15, 16]. It is a hemodynamic indicator that reflects backflow. Higher OSI means more backflow, which can cause the formation of vascular plaques and lesions. OSI can be calculated as follows:

1 $${\text{OSI}} = \frac{1}{2}\left( {1 - \frac{{\left| {\mathop \smallint \nolimits_{0}^{T} \overrightarrow {{\tau_{\omega } }} {\text{d}}t} \right|}}{{\mathop \smallint \nolimits_{0}^{T} \left| {\overrightarrow {{\tau_{\omega } }} } \right|{\text{d}}t}}} \right)$$

where *τ*_*ω*_ is WSS and *T* is the cardiac cycle. In contrast, the lower OSI is beneficial to benign remodeling of stenotic vessels. There are some areas in the cerebral arteries that have pronounced curves and a large angle of torsion, such as cerebral part of the internal carotid artery and the posterior communicating artery, among others. These tend to be the high incidence areas of cerebral artery plaques and aneurysms, as blood flow moves both in the anterograde and in the retrograde directions in the curved vessels, while OSI increases, which promotes the development of atherosclerosis [17]. In addition, wall shear stress gradient (WSSG) also affects the remodeling of the vascular endothelial layer. Positive WSSG inhibits both proliferation and apoptosis of vascular endothelial cells; negative WSSG promotes proliferation and apoptosis of cells [18]. Treatment effects of EECP acting on VECs are long-term hemodynamic effects. Both acute and long-term hemodynamic effects are primary mechanisms of EECP treatment for stroke patients.

Numerous clinical reports and animal experiments have demonstrated the hemodynamic effects of EECP on cerebral arteries. Xiong and Lin compared the velocity waveforms of middle cerebral artery flow in patients with stroke before and during counterpulsation. They found that diastolic blood flow of the cerebral artery significantly increased during counterpulsation [19–22]. Using an animal experiment, Zhang and colleagues observed that long-term application of EECP reversed the progression of high cholesterol and caused benign remodeling of cerebral arteries. Zhang concluded that WSS was the major factor for promoting restoration and remodeling [8]. These studies have shown that the hemodynamic effects of EECP were effective for the treatment of ischemic stroke disease. However, due to patients’ physiological differences, a phenomenon often occurs in which the same counterpulsation mode may result in different effects for different patients in clinical treatment [21]. This means that the counterpulsation mode should be appropriately adjusted for different stroke patients to optimize treatment. Based on the actual operation of clinical EECP equipment, the adjustable counterpulsation modes include pressure amplitudes and pressurization durations of cuffs wrapped around calves, thighs and buttocks. According to clinical surveys, EECP devices that have been manufactured by different companies may have differing modes of operation. Some EECP devices always maintain the same pressure amplitude for the three body parts, but pressure can be adjusted [23]. However, some devices only use one pressure amplitude and so apply the same pressure to the three parts. Therefore, for clinical treatment of stroke patients, three questions must be answered: (1) During counterpulsation, should the same pressure amplitude be applied to the three body parts? (2) How can pressure amplitude applied to each part be adjusted? (3) How can pressurization duration of counterpulsation be adjusted?

When focusing on the concerns of clinical applications, it is necessary to design a simple, rapid method to obtain responses for acute hemodynamic indicators and localized hemodynamic details of the cerebral arteries to EECP. This study initially used a geometric multiscale numerical 0D/3D model of the cerebral artery-blood circulatory system to explore hemodynamic effects of different counterpulsation modes on cerebral arteries. The geometric multiscale method is a special strategy that simulates the blood circulatory system. This method uses different models to simulate different parts of the circulatory system [24–26]. The three-dimensional (3D) model can be used to observe the hemodynamic environment of the cerebral artery with localized details, which determine long-term hemodynamic effects. The lumped parameter (0D) model could be used to simulate acute hemodynamic effects during the application of EECP. Characteristics of the geometric multiscale model mean it is suitable for hemodynamic simulation of EECP, as the localized hemodynamic details in the 3D model can observed in real time when counterpulsation is applied to the 0D model. The mean arterial pressure (MAP) and CBF, which are the clinical indicators commonly used to evaluate acute hemodynamic effects on patients with cerebral ischemic stroke, can be calculated using a 0D model, while the localized hemodynamic environment, including changes to WSS and OSI that significantly affect the long-term hemodynamic effects, can be observed with the 3D model.

This study aimed to establish a geometric multiscale method to explore acute and long-term hemodynamic effects on the cerebral artery caused by EECP. The effectiveness of our model was examined by comparing simulation results with clinical data. Following simulation of different counterpulsation modes, optimal strategies for EECP treatment mode were suggested for patients with cerebral ischemic stroke.

## Results

### Influence of the same and different pressure amplitudes of each part

**Fig. 1:**
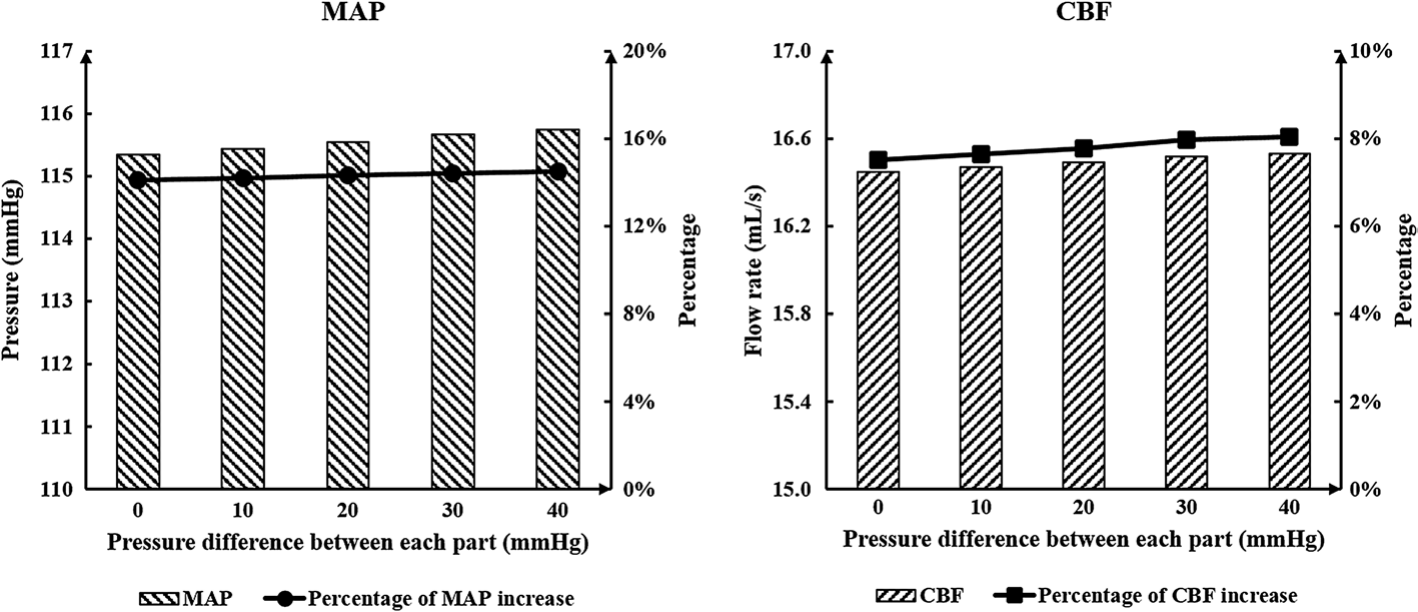
Calculated MAP and CBF of simulated experimental results of various pressure amplitude differences among the three body parts. MAP is mean arterial pressure and CBF is cerebral blood flow

MAP is the clinical indicator typically used to evaluate the acute effects on cerebral ischemic stroke, and CBF is the most direct indicator to reflect blood perfusion of cerebrovascular vessels. Both of these are acute hemodynamic indicators. To answer the clinical question about whether similar or different pressure amplitudes at calves, thighs and buttocks should be maintained, numerical simulations were conducted. Results of MAP and CBF, which can be seen in Table 1 and Fig. 1, show there was little difference between each experimental group. The acute hemodynamic indicators increased slightly as the pressure difference was increased for each body part.

**Table 1:** Variations of acute indicators between experimental and control groups

	Pressure of calves	Pressure of thighs	Pressure of buttocks	Pressure difference	MAP	CBF	Percentage of increase in MAP (%)	Percentage of increase in CBF (%)
Control group	0	0	0		85.08	12.10		
Experiment groups	200	200	200	0	115.34	16.45	35.57	35.95
	210	200	190	10	115.44	16.47	35.68	36.12
	220	200	180	20	115.54	16.49	35.80	36.28
	230	200	170	30	115.67	16.52	35.95	36.53
	240	200	160	40	115.75	16.53	36.05	36.61

Pressure unit: mmHg, flow rate unit: mL/s

*MAP* mean arterial pressure, *CBF* cerebral blood flow

### Influence of the pressure amplitudes of the three parts

**Fig. 2:**
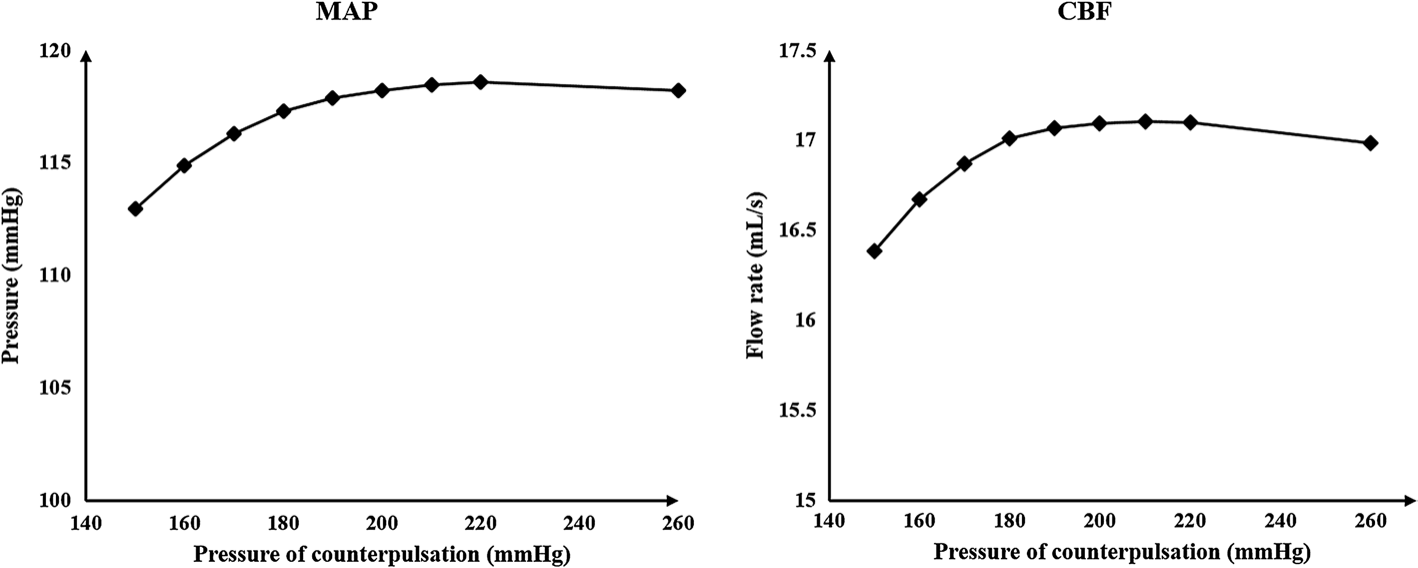
Calculated MAP and CBF of simulated experimental results of various pressure amplitudes. Same pressure amplitude was maintained in the three body parts. MAP is mean arterial pressure and CBF is cerebral blood flow

**Fig. 3:**
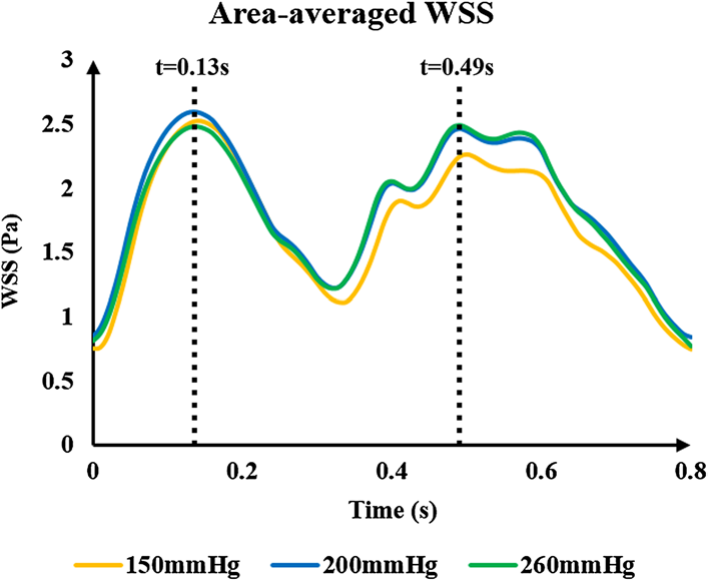
Area-averaged WSS waveforms of simulated experimental results of three pressure amplitudes. The same pressure amplitude was maintained in the three body parts; 0.13 s was the maximum time point during systolic phase, and 0.49 s was the maximum time points during diastolic phase. WSS is wall shear stress

**Fig. 4:**
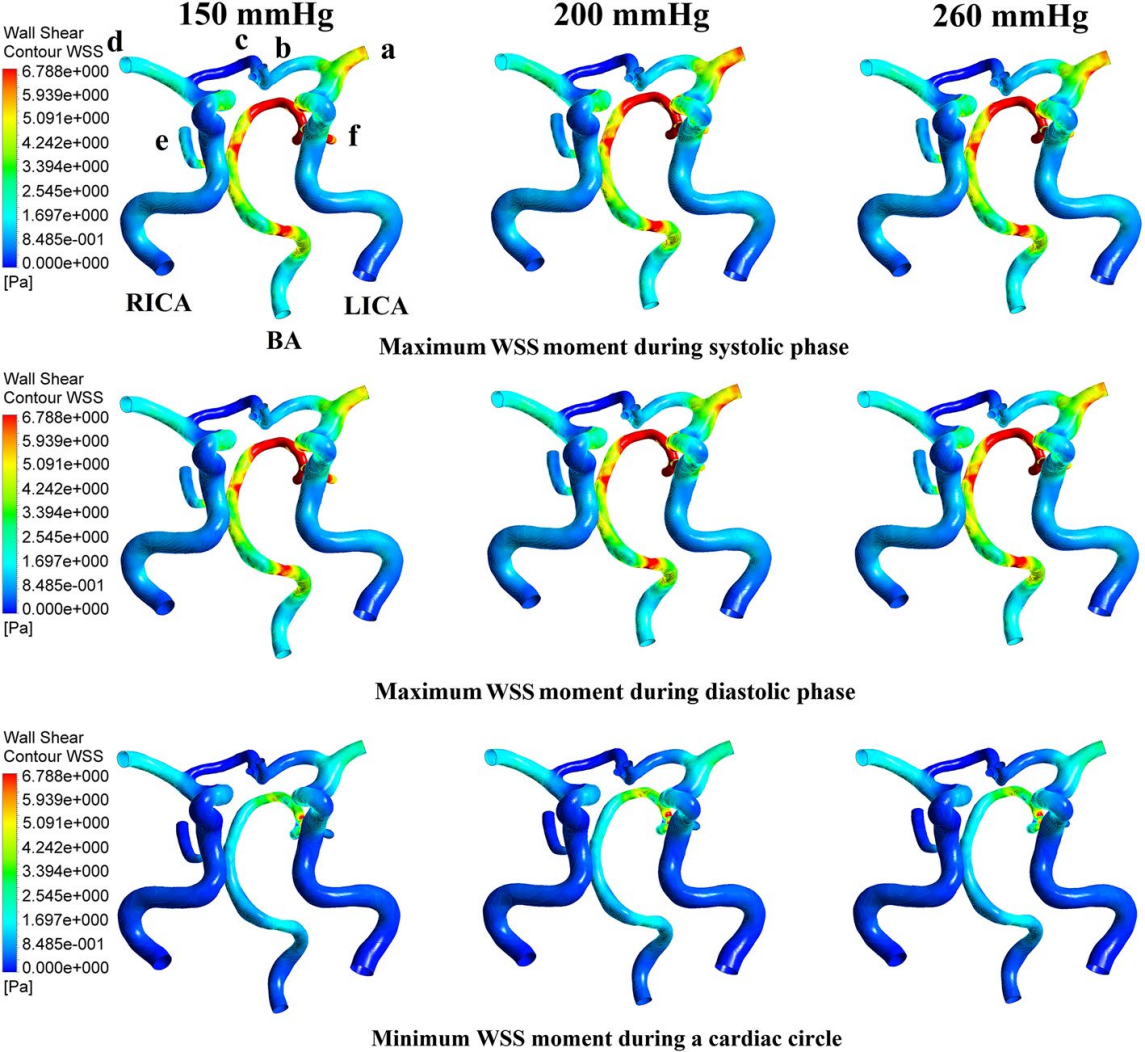
WSS contours of the cerebral artery at each extremum time points of the simulated experimental results of three pressure amplitudes. Extremum time points include the maximum WSS time point during systolic and diastolic phases as well as the minimum WSS time point during a cardiac circle. WSS is wall shear stress, RICA and LICA are right and left internal carotid arteries, respectively, BA is basilar artery, b and c are anterior cerebral arteries, a and d are middle cerebral arteries, and e and f are posterior cerebral arteries

It can be concluded from the above results that using both the same and different pressure amplitudes for each part resulted in nearly the same acute hemodynamic effects and thus caused almost the same long-term effects. Therefore, we conducted a series of numerical experiments with different pressure amplitudes while maintaining the same pressure in the three body parts. Calculated MAP and CBF values are shown in Fig. 2. Figure 3 demonstrates the simulation waveforms of the area-averaged WSS under pressure amplitudes of 150, 200 and 260 mmHg in the three body parts. The time points of maximum WSS during systole and diastole were 0.13 and 0.5 s, respectively, while the minimum time point during a cardiac circle was 0.0 s. WSS contours of the cerebral artery at each extremum time point are shown in Fig. 4. During systole, time-averaged WSS (TAWSS) under the three pressure amplitudes was 1.826, 1.875 and 1.839 Pa, while during diastole, TAWSS was 1.646, 1.818 and 1.843 Pa, respectively. These results suggest that when the pressure amplitude of the three parts was less than 200 mmHg, both MAP and CBF increased slightly with the increasing pressure amplitude. WSS had a very slight increase during diastole and almost no variation during systole despite increasing pressure amplitude. Finally, changes were not observed when the pressure amplitude was greater than 200 mmHg.

### Influence of the pressurization durations of the three parts

**Fig. 5:**
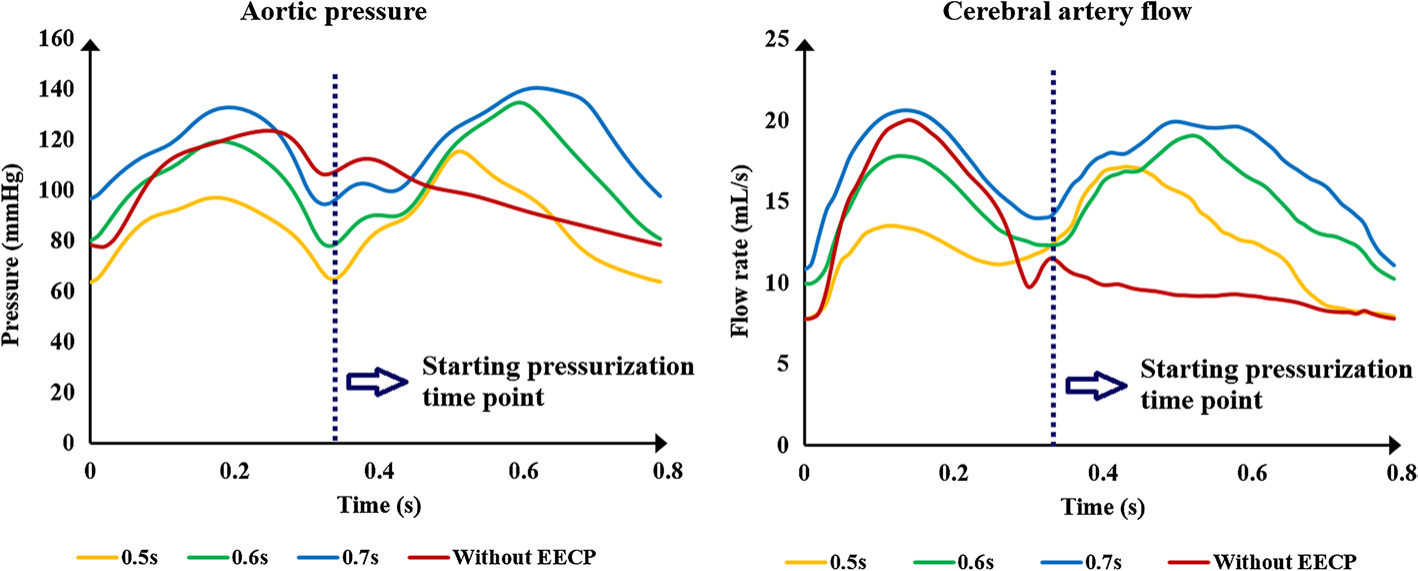
Aortic pressure and CBF waveforms of simulated experimental results of three pressurization durations and waveform without EECP. Pressurization durations were determined by pressure release time points (0.5, 0.6 and 0.7 s) and the pressure amplitudes of the three body parts were 200 mmHg. CBF is cerebral blood flow and EECP is enhanced external counterpulsation

**Fig. 6:**
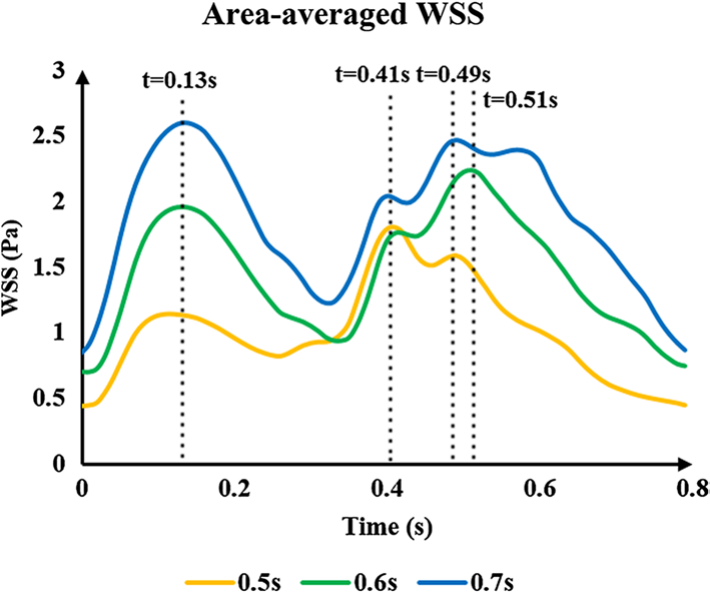
Area-averaged WSS waveforms of simulated experimental results of three pressurization durations. Pressurization durations were determined by pressure release time points (0.5, 0.6 and 0.7 s), while pressure amplitudes of the three body parts were 200 mmHg. 0.13 s was the maximum time point during systolic phase under three pressure release time points, 0.41, 0.51 and 0.49 s were maximum time points during diastolic phase under the pressure release time points of 0.5, 0.6, 0.7 s, respectively. WSS is wall shear stress

**Fig. 7:**
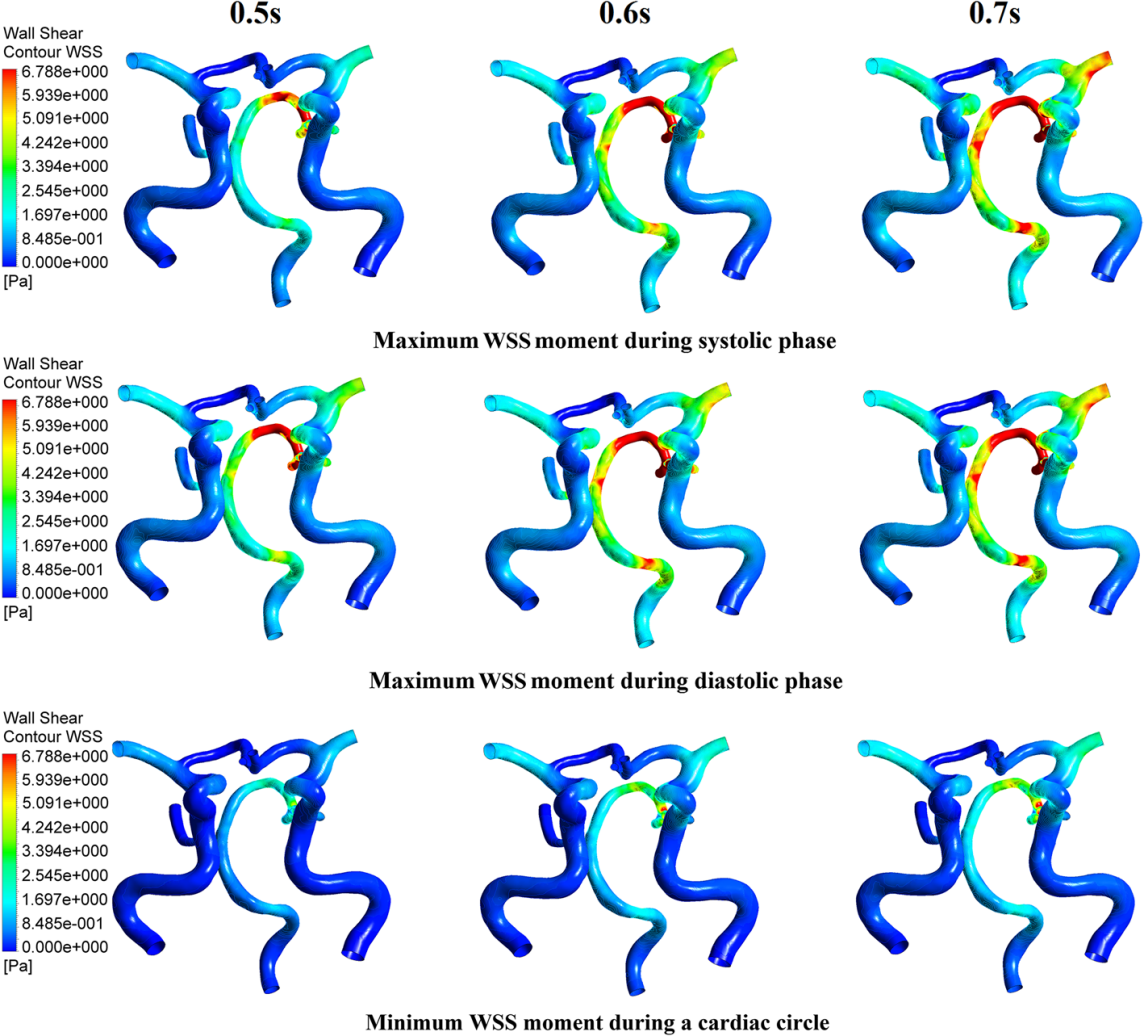
WSS contours of cerebral artery at each extremum time points of the simulated experimental results of three pressurization durations. Extremum time points include maximum WSS time point during systolic and diastolic phases, and minimum WSS time point during a cardiac circle. WSS is wall shear stress

**Fig. 8:**
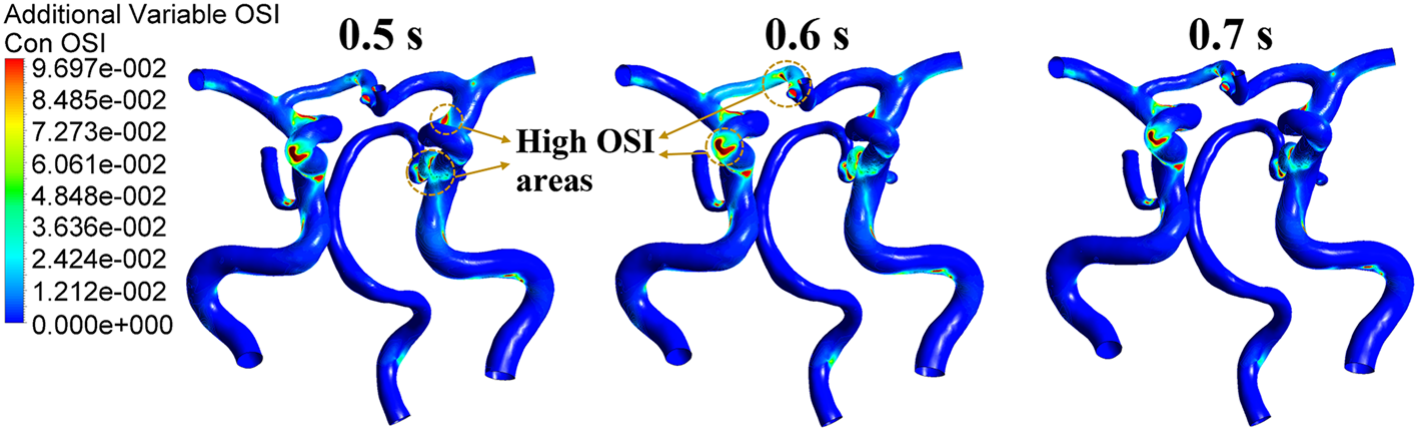
OSI contours of the cerebral artery during a cardiac circle under three pressurization durations. OSI is oscillatory shear index

Pressurization duration is a parameter that influences treatment adequacy. Pressurization duration depends on pressure release time point. The simulation waveforms of aortic pressure and CBF under different pressure release time points are shown in Fig. 5. Mean values of MAP, CBF and TAWSS during a cardiac circle are displayed in Table 2, where TAWSS is the mean value of area-averaged WSS during a cardiac circle. These results show a significant improvement of hemodynamic indicators. The simulation waveforms of area-averaged WSS are shown in Fig. 6. For three pressurization durations, it could be observed that the time point of maximum WSS during systole was 0.13 s, the time points of maximum WSS during diastole were 0.41, 0.51 and 0.49 s, respectively, and the minimum time point during a cardiac circle was 0.0 s. WSS contours of the cerebral artery at each extremum time point are shown in Fig. 7. Similarly, the WSS in cerebral artery increased significantly as the pressurization duration increased. The highest WSS in cerebral artery for both systole and diastole was observed for the mode of pressure release at 0.7 s. In addition, effects of different pressurization durations on OSI are shown in Fig. 8. According to theory [27], the threshold for distinguishing high and low mean OSI is 0.02. As a result, sizes and mean values of high OSI areas (OSI > 0.02), as shown in Fig. 8, were extracted. The total area size of the cerebral arteries was 5072.6 mm^2^, while sizes of high OSI areas under the three pressurization durations were 376.6, 415.4 and 314.8 mm^2^, which were 7.42%, 8.19% and 6.21% of the total size. The mean values of high OSI area under the three pressurization durations were 0.061, 0.063 and 0.049, respectively. The above data demonstrate that when pressure releases at 0.7 s during a cardiac circle, both the size and mean value of high OSI area in the cerebral arteries show maximum reduction. Finally, inlet velocity and Reynolds number at the highest flow time point (0.13 s during the cardiac circle) for the mode of pressure release at 0.7 s were presented to examine the rationality of simulation, as the maximum flow appears at this counterpulsation mode. The velocities of three inlets were 0.65, 0.74 and 0.21 m/s, while the Reynolds numbers were 979.74, 995.69 and 219.68, respectively. Both of these factors were in the reasonable range.

**Table 2:** Mean values of MAP, CBF and time-averaged WSS (TAWSS) during a cardiac circle under three pressure release time points

Pressure release time point	MAP	CBF	TAWSS
0.5	86.357	12.239	1.012
0.6	105.032	14.934	1.449
0.7	118.221	17.128	1.869

Unit: time point: s, pressure: mmHg, flow rate: mL/s, WSS: Pa

*MAP* mean arterial pressure, *CBF* cerebral blood flow, *WSS* wall shear stress

## Discussion

### Re-thinking about hemodynamic responses to different counterpulsation modes

**Fig. 9:**
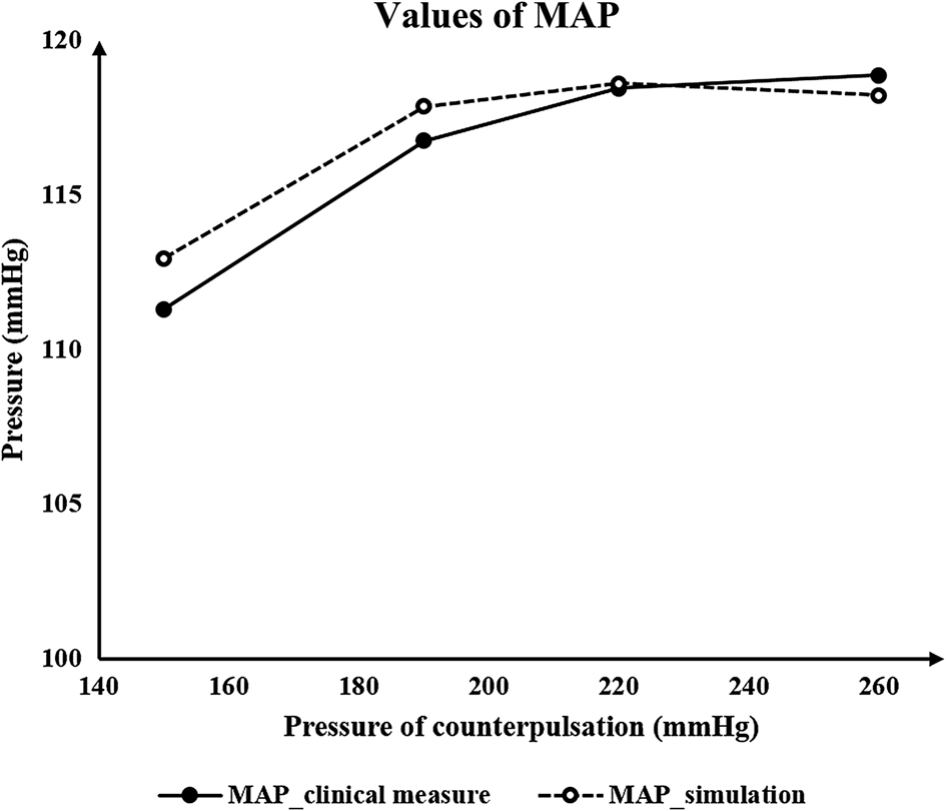
Comparison of the values of MAP between simulation results and the clinical data. MAP is mean arterial pressure

When addressing the aforementioned clinical questions about the hemodynamic effects of different counterpulsation modes for patients with cerebral ischemic stroke, it can be concluded from the above results that using the same and different pressure amplitudes for each part resulted in nearly the same acute hemodynamic effects, in turn leading to the same long-term hemodynamic effects. Thus, it may not be necessary to adopt different pressure amplitudes for each body part in clinical operation of EECP. In addition, as shown in the results described in “Limitations” section, hemodynamic effects hardly changed when pressure amplitude was greater than 200 mmHg as vascular collapse occurred in the external iliac artery, meaning that it was difficult for an even greater pressure to change the blood flow. As a result, it can be concluded that an increase in pressure amplitude may result in a slight improvement of treatment effects for stroke patients. Similar research has been conducted in clinical settings. Lin [23] used different pressure amplitudes to observe acute treatment effects for stroke patients and recorded MAP under each pressure. A comparison between our results and that clinical data is shown in Fig. 9. The relative errors of the points under each pressure were 1.47, 0.95, 0.13 and 0.56%, respectively. This small difference explains the accuracy of our calculations as well as the effectiveness of the model.

Differing from hemodynamic influence of pressure amplitude, the pressurization duration significantly impacted both acute hemodynamic effects and localized details. Nevertheless, as WSS and OSI have a substantial impact on benign remodeling of blood vessels during EECP, the calculation of WSS and OSI is more crucial than acute physiological indicators. According to the functional theory of VECs and local hemodynamic WSS [12], the proper physiological range of long-term WSS for VECs is 1–7 Pa. WSS is not beneficial to atherosclerosis when it is less than 1 Pa, and could damage VECs when greater than 7 Pa. As can be seen in Table 2, when the pressurization duration of the counterpulsation mode was based on the 0.5-s pressure release time point, the TAWSS was 1.012 Pa, which is very close to 1 Pa. As a consequence, the short pressurization duration had little treatment effect for cerebral ischemic stroke if there was a stenosis. In addition, when pressurization duration was based on the 0.7-s pressure release time point, the TAWSS of 1.869 Pa was less than 7 Pa, which did not damage the VECs.

Aside from WSS, blood flow characteristics are also key factors which influence the phenotype of vascular endothelial cells and promote atherosclerosis. Taylor [28] has reported that reducing flow oscillations, increasing WSS and reducing shear stress oscillations benefit atherosclerotic plaque and also that OSI is the indicator which reflects the flow characteristic of quantified oscillations in shear stress. Results in Fig. 8 and the variation of high OSI areas suggest that the maximum reduction of OSI caused by the 0.7-s pressure release time point will benefit the vascular endothelium. This means that during the long pressurization duration, the increase in WSS and decrease in OSI are the crucial factors for inhibiting the development of atherosclerosis. In summary, pressurization duration could be lengthened to achieve possible sufficient treatment effects in clinical operation, but the pressure should not be released too late to avoid influencing normal cardiac ejection in the subsequent cardiac cycle.

Our previous study explored acute hemodynamic responses to different counterpulsation modes [29]. We found that high pressure amplitude of thighs could result in the increase in SBP and DBP, thus increasing MAP and promoting better treatment. While, in the previous study, the critical pressure value for vascular collapse was not specified, here, we presented a specified pressure value of 200.668 mmHg for vascular collapse of external iliac artery. As a result, hemodynamic effects hardly changed when the pressure amplitude was greater than 200 mmHg as vascular collapse occurred in the external iliac artery. It can be observed from Fig. 2 that when the pressure amplitude was lower than 200 mmHg, the mean arterial pressure and cerebral blood flow showed some improvement with increasing counterpulsation pressure. However, the hemodynamics showed only a small change when the pressure amplitude was over 200 mmHg, which is not specified in the previous study. Physiologically speaking, hemodynamics will not always be improved as pressure amplitude keeps increasing. Therefore, this finding is an update to those of the previous study.

### Limitations

This study has some limitations. In this paper, a series of numerical simulations were conducted without verification of clinical experiments. Although the parameters in the model were adjusted according to clinical experimental results, clinical studies should be carried out to verify the quantitative conclusions. Since WSS can be calculated by flow velocity and diameter of the vessels, quantitative WSS can be measured by transcranial Doppler (TCD) [19] for verification. Beyond that, some idealized models and hypotheses were presented in the current study. The fluid simulation was based on the rigid wall assumption and Newtonian flow assumption, while the models for the calculation of critical pressure value of vascular collapse were highly idealized. Although the cerebral arteries are small, there will be a gap between assumptions and reality. In future work, the fluid–structure coupling method could be adopted to simulate a physiological situation which is closer to reality, and more indicators should be proposed to simulate the complex remodeling effects of the blood vessels as comprehensively as possible. In addition, some numerical simulation experiments could be performed to calculate a more accurate critical pressure value of vascular collapse.

In addition, only one model of cerebral artery was used in this study. In order to acquire the conclusion that is suitable for most patients, more CTA images should be collected and more models reconstructed for the hemodynamic simulation. As the physiological structure of cerebral arteries is highly similar, simulation results for most patients may not differ greatly. However, this needs to be verified by more calculation.

Results from the current study provided a general rather than individual treatment strategy for most stroke patients. This means that the same counterpulsation mode may have a different impact on the CBF of patients with different anatomical physiology structures (such as different degrees of cerebral artery stenosis). Increased CBF can increase the WSS of the entire cerebral blood vessels but improving the WSS in the infarcted territories after different degrees of stenosis in different way [30]. Beyond that, due to differences in physiological parameters such as blood pressure, patients may have differing hemodynamic responses to the same counterpulsation mode. This means that it is necessary to develop a patient-specific strategy for EECP treatment. There is a need for more clinical data to develop a patient-specific algorithm, while individual simulations could be conducted to achieve the best treatment strategy.

## Conclusions

This study established a geometric multiscale model to research the hemodynamic effects of EECP on the cerebral artery while considering vascular collapse and cerebral autoregulation. Based on this model, acute variations in blood flow, blood pressure and localized hemodynamic details of cerebral artery could be observed. We suggest that when EECP is applied to patients with cerebral ischemic stroke, it may not be necessary to adopt different pressure amplitudes for the three parts. The increasing pressure amplitude of the three body parts may improve treatment effects slightly and will not benefit patients when it is over almost 200 mmHg. During counterpulsation, pressurization duration could be increased during the cardiac circle for the possible superior treatment outcomes. A short pressurization duration (0.5 s) may have poor treatment effects for stroke patients.

## Materials and methods

### Establishment of geometric multiscale model

Establishment of the 3D model was based on computed tomography angiography (CTA) images of the cerebral artery of a volunteer. Images were provided by The Eighth Affiliated Hospital, Sun Yat-sen University. Since the aim of this study was to investigate acute and long-term hemodynamic effects of different counterpulsation modes on cerebral arteries, the method utilized should be suitable for most patients. In addition, the model of the cerebral artery should, methodologically speaking, be representative of most patients. Therefore, a natural model without stenoses was chosen for reconstruction. Cerebral arteries were reconstructed based on CTA images. The 3D geometry of cerebral arteries was generated by Mimics and smoothed by Freeform, a touch-based interactive tool for the 3D geometry editing.

**Fig. 10:**
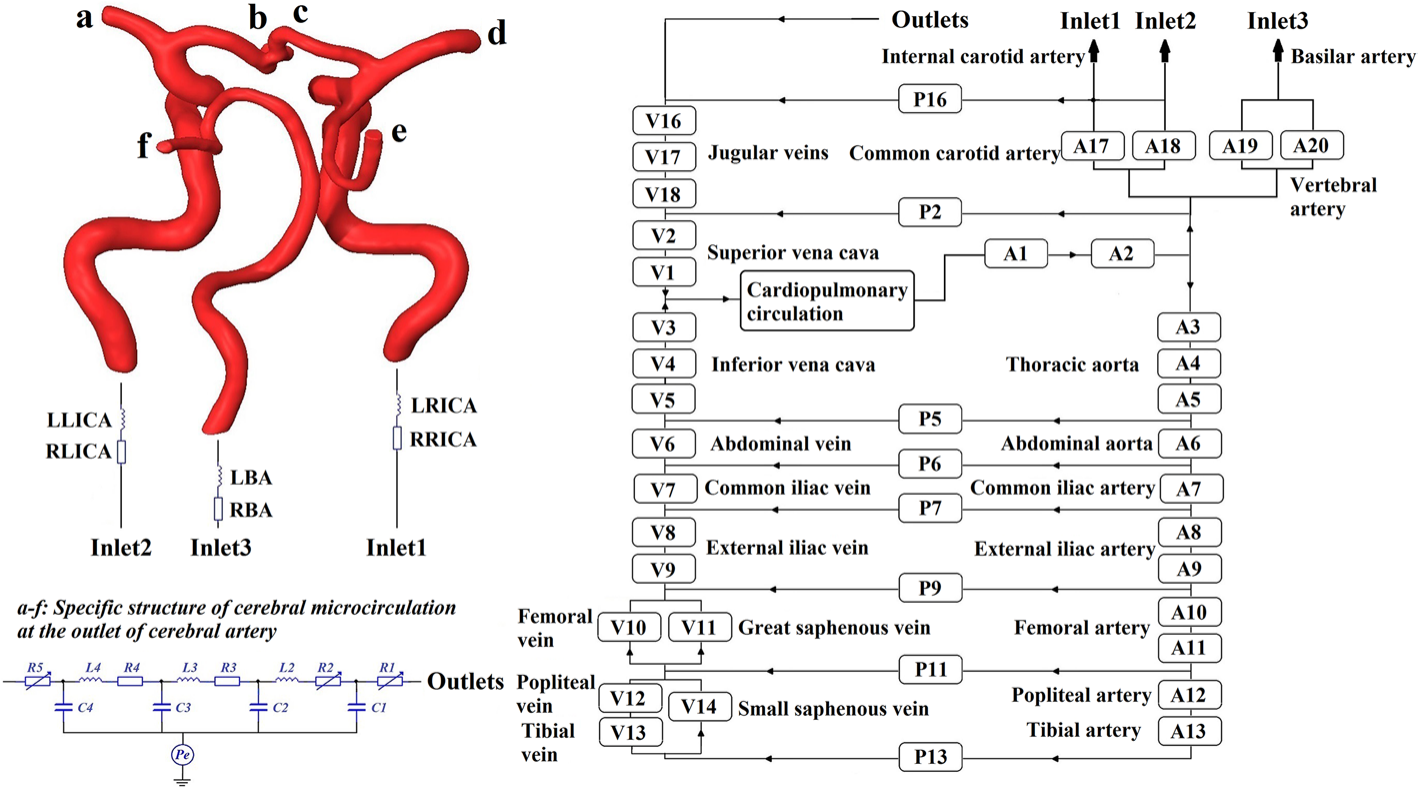
Geometric multiscale model of the cerebral artery with three inlets and six outlets. At the inlet of the 3D model, the 0D model of right and left internal carotid arteries (RICA, LICA) and the basilar artery (BA) were coupled; at the outlet of the 3D model, anterior cerebral arteries (b, c), middle cerebral arteries (a, d) and posterior cerebral arteries (e, f) were coupled

**Fig. 11:**
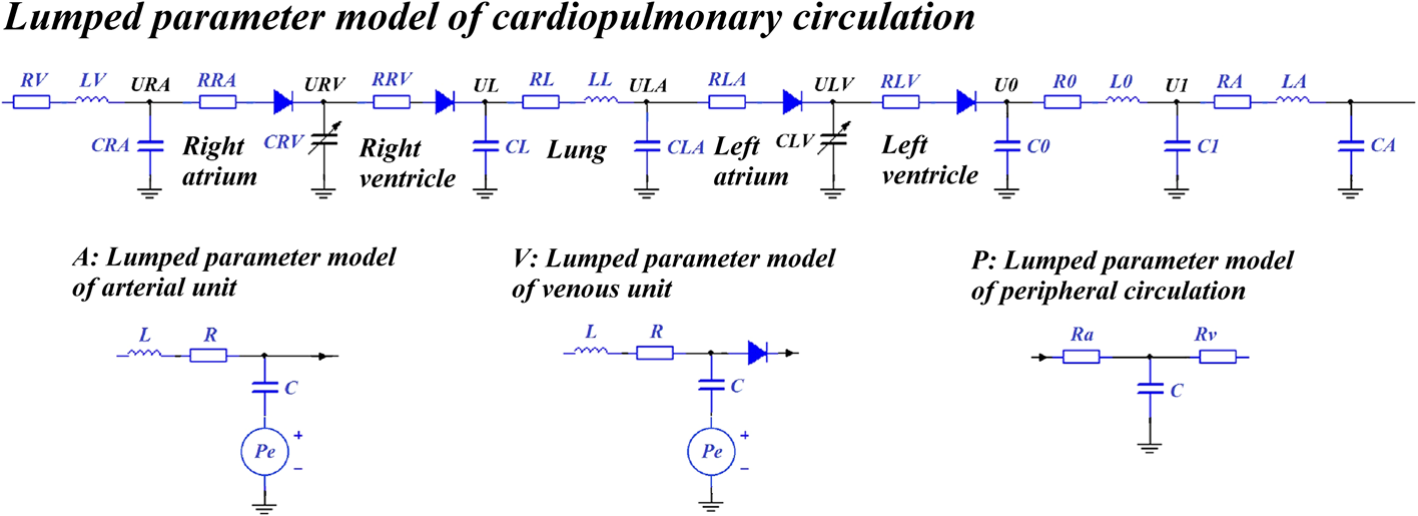
Detailed structures of the whole blood circulatory system. The voltage sources Pe in artery and vein units were used to simulate the pressure provided by the counterpulsation cuffs, which only exist in A8–A13 and V8–V13. Diodes in V8–V13 were used to simulate the lower limbs’ venous valve

Establishment of the 0D model was based on 3D reconstruction results. Lumped parameter modeling is a common method which utilizes circuit elements to simulate the blood circulatory system. The 0D model is often coupled to the inlet and outlet of the 3D model as a boundary condition in a geometric multiscale model. Following previous studies [31–33], we established a complete, closed-loop 0D model for the systemic simulation as shown in Fig. 10. This model had 17 artery and vein units, 8 peripheral circulation units and a cardiopulmonary circulation unit. The detailed structures of the whole blood circulatory system can be seen in Fig. 11. Existing research [33] has outlined the parameters of the 0D model. Based on these parameters, the value of each circuit element in our model was adjusted to match classic physiological waveforms and clinical measurements. Parameter values are shown in Tables 3 and 4.

**Table 3:** Parameters of the blood circulatory system

Serial number	Arterial unit			Venous unit			Peripheral circulation		
	*R*	*C*	*L*	*R*	*C*	*L*	Ra	Rv	*C*
1	0.00426	0.1050	0.00035	0.03780	0.0800	0.00050			
2	0.00395	0.1050	0.00020	0.03860	0.0950	0.00051	1.50	2.60	0.00105
3	0.00405	0.1067	0.00012	0.05490	0.0200	0.00008			
4	0.00695	0.1041	0.00010	0.05560	0.0200	0.00008			
5	0.00897	0.0350	0.00010	0.06030	0.0280	0.00010	2.36	2.34	0.00520
6	0.00933	0.0240	0.00023	0.08080	0.0101	0.00018	3.33	2.96	0.00103
7	0.00848	0.0168	0.00022	0.12000	0.0100	0.00014	4.18	3.55	0.00108
8	0.01000	0.3050	0.02275	0.20000	0.0900	0.00521			
9	0.01200	0.0150	0.02275	0.19000	0.0300	0.01187	5.30	4.20	0.00100
10	0.12800	0.3620	0.03528	0.51000	0.0400	0.01581			
11	0.15800	0.0520	0.03500	0.53000	0.0500	0.01855	5.40	4.70	0.00100
12	0.17500	0.0520	0.00514	1.42000	0.0200	0.03135			
13	0.19100	0.0510	0.00514	1.41000	0.0100	0.04089	6.05	5.35	0.00010
14				1.41000	0.0100	0.04089			
16				0.18000	0.0600	0.00040	4.40	4.70	0.00168
17	0.02535	0.0150	0.00015	0.18000	0.0700	0.00035			
18	0.02518	0.0150	0.00015	0.16000	0.0070	0.00030			
19	0.02535	0.0100	0.00020						
20	0.02518	0.0100	0.00020						

Unit: *R*: mmHg s/mL, *C*: mL/mmHg, *L*: mmHg s^2^/mL

*R*: resistance, *C*: capacitance, *L*: inductance, Ra: arterial microcirculatory resistance, Rv: venous microcirculatory resistance

**Table 4:** Parameters of cardiopulmonary circulation and inlet of 3D model

*R*		*C*		*L*	
RV	0.10271	CRA	0.5946	LV	0.00012
RRA	0.00353	CL	0.3436	LL	0.00021
RRV	0.00023	CLA	2.5000	L0	0.00150
RL	0.00688	C0	0.1500	LA	0.00070
RLA	0.00701	C1	0.1950	LLICA	0.15000
RLV	0.50300	CA	0.1800	LRICA	0.15000
R0	0.03771			LBA	1.12000
RA	0.00251				
RLICA	0.05030				
RRICA	0.05010				
RBA	0.04030				

Unit: *R*: mmHg s/mL, *C*: mL/mmHg, *L*: mmHg s^2^/mL

Establishment of the geometric multiscale model of the cerebral artery was based on the 0D and 3D models. Based on the physiological structure of the 3D model of the cerebral artery, the coupling interface of the geometric multiscale model was designed to align with the internal carotid artery, basilar artery and brain microcirculation [34]. Utilizing a coupling algorithm [35], the geometric multiscale model of the cerebral artery was developed, as shown in Fig. 10. In the coupling algorithm, the 0D model calculates the inlet flow and outlet pressure as the boundary conditions for the 3D model calculation, while the inlet pressure and outlet flow calculated by the 3D model are provided for missing values in the 0D model calculation. The data interaction between the 0D model and 3D model follows these formulas:

2 $$\bar{P}_{{3{\text{D}},{\text{in}}}} = \frac{1}{{A_{{3{\text{D}},{\text{in}}}} }}\mathop \int \nolimits_{{\tau_{\text{in}} }}^{{}} P{\text{d}}\tau = P_{{0{\text{D}},{\text{in}}}}$$

3 $$Q_{{3{\text{D}},{\text{out}}}} = \rho \mathop \int \nolimits_{{\tau_{\text{out}} }}^{{}} \mu n_{i} {\text{d}}\tau = Q_{{0{\text{D}},{\text{out}}}}$$

where $\bar{P}_{{3{\text{D}},{\text{in}}}}$ is the mean inlet pressure calculated by the 3D model, $A_{{3{\text{D}},{\text{in}}}}$ is the inlet area of the 3D model, $\tau_{\text{in}}$ is integral domain (the inlet plane of the 3D model), $P$ is the pressure of each element on the inlet plane of the 3D model, ${\text{d}}\tau$ is the differential area element, $P_{{0{\text{D}},{\text{in}}}}$ is the missing value of the 0D model, which is the mean inlet pressure of the 3D model, $Q_{{3{\text{D}},{\text{out}}}}$ is the outlet flow calculated by the 3D model, $\rho$ is blood density, $\tau_{\text{out}}$ is integral domain (the outlet plane of the 3D model), $\mu$ is the node velocity of the outlet plane of the 3D model, $n_{i}$ is the normal vector of the outlet plane and $Q_{{0{\text{D}},{\text{out}}}}$ is the missing value of the 0D model (the outlet flow of the 3D model). The inlet of the 3D model was coupled to the internal carotid artery and basilar artery, while the outlet of the 3D model (a–f) was coupled to the cerebral microcirculation. Specific structures and parameters of the cerebral microcirculation at the outlet of the cerebral artery have previously been described [34].

### Hemodynamic calculation details of the geometric multiscale model

Hemodynamic calculation of the 3D model was conducted with fluid simulation software ANSYS-CFX. Fluid density was 1050 kg/m^3^, viscosity was 0.0035 Pa/s, the number of fluid elements was 1,186,933, the vessel wall was simplified to a rigid wall and blood flow was transient. In addition, local blood flow was considered to be performed at a constant temperature, ignoring the change in heat, while the energy conservation equation was disregarded. Therefore, pulsating blood flow in the cerebral artery is a transient incompressible Newtonian fluid flow problem.

The Navier–Stokes equations were applied for hemodynamic simulations of the 3D model, and the flow was assumed to be laminar. Discretization in time was based on second-order backward Euler and an implicit scheme. During multiscale calculation, the time step of 3D model was 0.001 s, while the time step of 0D model was 0.00001 s. The two models achieved a data exchange after 100 times calculation of 0D model. The continuous computational domain was divided into finite discrete sets, which were mesh nodes, while discretization in space was based on divided mesh nodes. The differential equations and their solutions on these mesh nodes were transformed into corresponding algebraic equations, meaning that discrete equations were established. Discrete equations were solved, and the solution on each node could be acquired. In addition, approximate solutions between nodes were considered to be a smooth variation, while an interpolation method was used to obtain approximate solutions for the entire computational domain.

The heart module is a key source of power for the entire circulatory system. Ventricular systolic and diastolic function can be reflected by the pressure–volume relationship of ventricles. With the same ventricular volume variation, greater ventricular contraction pressure indicates a stronger systolic heart function. A time-varying function *E*(*t*) that can reflect both the systolic and the diastolic functions of the ventricle was used in the heart module to simulate ventricular contraction. The function *E*(*t*) can be described by the ventricular pressure–volume relationship, as follows [36]:

4 $$E\left( t \right) = \frac{{P_{\text{sv}} \left( t \right)}}{{V_{\text{sv}} \left( t \right) - V_{0} }}$$

where *P*_sv_(*t*) is the time function of ventricular pressure (mmHg), *V*_sv_(*t*) is the time function of ventricular volume (mL) and *V*_0_ is the ventricular reference volume (mL), a theoretical volume relative to “zero ventricular pressure.” Application of ventricular contraction function *E*(*t*) to the variable capacitances of both left (*CLV*(*t*)) and right ventricles (*CRV*(*t*)), as shown in Fig. 11, produced a pulse wave on *C0* which acted as an energy source. Mathematically, one could fit Eq. (4) using the following approximation to describe the ventricular systole function:

5 $$E\left( t \right) = \left( {E_{ \text{max} } - E_{ \text{min} } } \right) \cdot E_{n} \left( {t_{n} } \right) + E_{ \text{min} }$$

where *E*_*n*_(*t*_*n*_) is a double hill function, as follows [37]:

6 $$E_{n} \left( {t_{n} } \right) = 1.55\left[ {\frac{{\left( {\frac{{t_{n} }}{0.7}} \right)^{1.9} }}{{1 + \left( {\frac{{t_{n} }}{0.7}} \right)^{1.9} }}} \right]\left[ {\frac{1}{{1 + \left( {\frac{{t_{n} }}{1.17}} \right)^{21.9} }}} \right]$$

where *t*_*n*_ is *t*/*T*_max_, and *T*_max_ has a linear relationship with the personalized cardiac cycle *t*_c_ (0.8 s) as follows:

7 $$T_{ \text{max} } = 0.2 + 0.15t_{\text{c}}$$

**Fig. 12:**
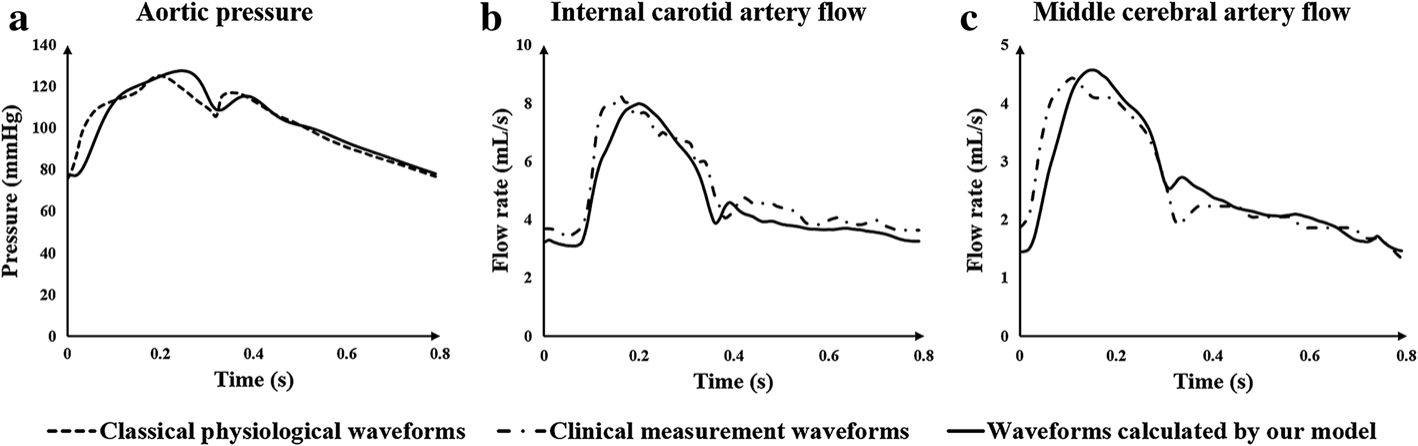
Effectiveness validation of the model without applying counterpulsation. **a** Comparison of aortic pressure between the classical physiological and simulated waveforms calculated by our model, while (**b**) and (**c**) are the comparison of internal carotid artery flow and middle cerebral artery flow between the clinical measurement and simulated waveforms

Values of *E*_max_ and *E*_min_ significantly impact the aortic pressure and cardiac output. *E*_max_ and *E*_min_ values for left and right ventricles were determined differently due to their different systolic strengths. Combined with the physiological data of most patients, it was determined that *E*_max_left_ was 6.0, *E*_min_left_ was 0.012, *E*_max_right_ was 0.00042, and *E*_min_right_ was 0.00003. Using the above methods and parameters, physiological waveforms were calculated. Comparisons between classical physiological waveforms, clinical measurement waveforms and waveforms calculated by our model are shown in Fig. 12. According to clinical reports, the total CBF is approximately 15–20% of cardiac output [38]. The CBF is fed by both internal carotid arteries and vertebral arteries, while the flow rate of internal carotid arteries tends to be three times the vertebral artery flow [39]. In our model, the calculated internal carotid artery flow is 9.1 mL/s, the vertebral artery flow is 3 mL/s, and the total CBF is 12.1 mL/s, 15.3% of cardiac output. This small difference in numerical values and waveforms between classical and simulation results supports the practicability of our model.

**Fig. 13:**
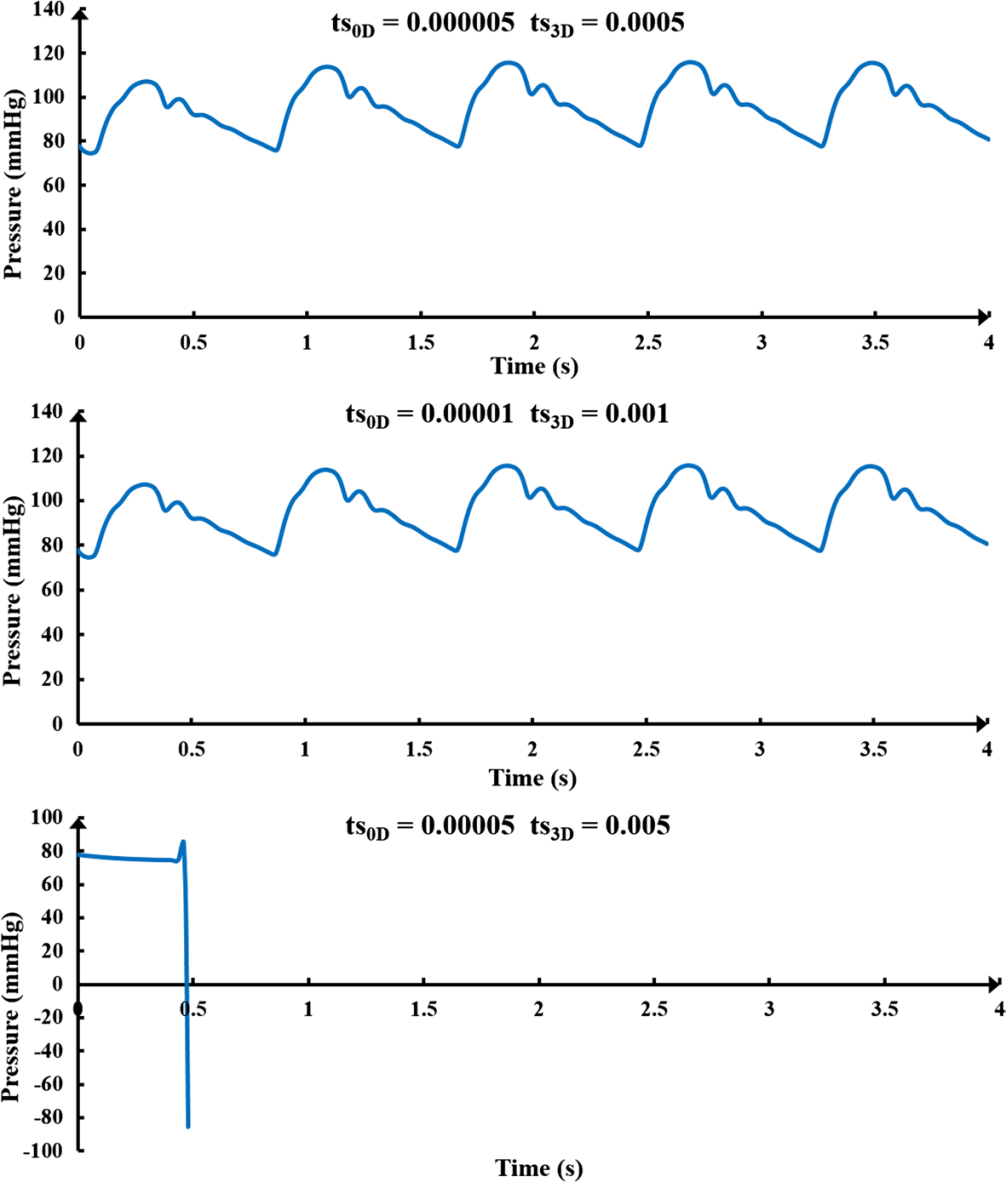
Time step test results. The ts_0D_ is the time step of 0D model (lumped parameter model) and the ts_3D_ is the time step of 3D model

Since the multiscale model in this study was a closed-loop, huge and complex model coupling by cerebral artery and blood circulatory system, the calculation cannot be convergent through the use of rough mesh or bigger time step. The time step of the 3D and 0D models was optimized to decrease the calculation time, while attaining convergence. As a result, a steady-state analysis of mesh dependency by aiming at WSS and CBF with constant pressure boundary conditions was conducted, as shown in Table 5. The time step tests aiming at aortic pressure can be seen in Fig. 13. Test results ensured that the mesh size (1,186,933 fluid elements) and time step chosen in this study (ts_0D_ was 0.00001 and ts_3D_ was 0.001) were optimal and that calculation results were credible.

**Table 5:** Steady-state analysis of mesh dependency for 3D model of cerebral artery by aiming at WSS and CBF with constant pressure boundary conditions

Size		Boundary conditions		CBF	Differences percentage	Area-averaged WSS	Differences percentage
Nodes	Elements	Inlet pressure	Outlet pressure				
316,652	394,380	90.2	85.2	18.053		1.699	
590,411	798,195	90.2	85.2	17.425	0.0348	1.608	0.0536
919,703	1,186,933	90.2	85.2	17.290	0.0077	1.596	0.0075

Pressure unit: mmHg, flow rate unit: mL/s, WSS unit: Pa

*CBF* cerebral blood flow, *WSS* wall shear stress

### Application of EECP

Application of pressure was based on four different parameters: inflation and deflation times, inflation time point, pressurization duration and pressure amplitude. When combined with the clinical operation, inflation and deflation times were set as 5 ms, following a previous study [31]. The inflation time point means the start pressurization time point of counterpulsation cuffs during the cardiac cycle. Based on the clinical operation, the inflation time point of the cuffs of the EECP equipment was triggered by the R-wave of electrocardiogram, which was the starting point of systole during a cardiac circle. After a systolic delay, which is approximately 0.25 s, cuffs were sequentially inflated. As a result, the inflation time point for calves in this study was set as 0.25 s during a cardiac circle. Based on clinical experience, EECP should be applied in a sequential manner and the interval between each part should be 0.05 s [40]. Therefore, inflation time points for calves, thighs and buttocks were 0.25, 0.30 and 0.35 s, respectively. Differing from the inflation time point as well as inflation and deflation times, selections of the pressurization duration and pressure amplitude should be carefully considered as they determine the different treatment effects of counterpulsation modes. Following inflation time points, inflation and deflation times were determined, and hemodynamic indicators, including MAP, CBF and WSS, were calculated under different pressure amplitudes and pressurization durations for each of the body parts to investigate the hemodynamic effects of different counterpulsation modes, where pressure amplitude was in the clinical range [41]. Our previous study has presented the control chart of the counterpulsation mode [29]. In this study, in order to examine both acute and long-term hemodynamic effects, a series of numerical simulations were conducted to answer the clinical queries about optimal counterpulsation strategies.

In order to determine whether similar or different pressure amplitudes at the calves, thighs and buttocks should be maintained, comparison experiments were carried out with the 0.65-s pressure release time points during a cardiac circle of the three body parts. Five groups with unequal pressure differences between each part were the experimental group, and a group without application of EECP was the control group. According to the general pressure application method, the order of pressure amplitudes of the three parts tends to be that calf pressure is greater than or equal to thigh pressure, while thigh pressure is greater than or equal to buttock pressure [42].

To determine optimal pressure amplitudes and pressurization durations, different counterpulsation modes were applied to investigate hemodynamic responses. In the clinical operation, cuffs wrapped around the three parts usually release at the same time point. As a result, once inflation time points were determined, pressurization duration depended on the pressure release time point of the three body parts. Based on the 0.7-s pressure release time points during a cardiac circle of those parts, a series of pressure amplitudes (150–260 mmHg) was applied to observe hemodynamic variations of the cerebral artery. In addition, with the 200-mmHg pressure amplitude of each part, three pressure release time points (0.5, 0.6 and 0.7 s) during a cardiac circle were applied to explore the hemodynamic influence of pressurization duration. Hemodynamic indicators, including MAP, CBF, and WSS, were compared to evaluate treatment effects. It should also be noted that for a cardiac circle of 0.8 s, the pressure release time point was not more than 0.7 s to avoid the danger of influencing the normal cardiac ejection in the subsequent cardiac cycle. This is because when the pressure is released, it takes some time for the blood to perfuse into the lower body.

### Vascular collapse during counterpulsation

Vascular collapse is a classic vessel instability issue under external pressure. During EECP, arteries in the lower body are compressed by the cuffs. If the pressure amplitude is greater than a critical value, vascular collapse occurs, and the arteries will close. However, the critical value for vascular collapse of each artery in the lower body has yet not been determined. The critical pressure value of vascular collapse is the sum of pressure inside the blood vessel and the external pressure required for vascular instability. To achieve calculation of the threshold value, the vessel type must first be determined. By assuming that a blood vessel is a standard cylindrical vessel, different parts of the arteries in the lower body were characterized as either long cylindrical vessels or short cylindrical vessels, according to length, thickness and internal diameter. When the length of a vessel exceeded a critical value, that vessel was considered a long cylindrical vessel. Otherwise, it was considered a short cylindrical vessel. The formula for calculating the critical length is [43]:

8 $$L_{\text{cr}} = 1.17D\sqrt {\frac{D}{{\delta_{e} }}}$$

where *D* is the internal diameter of the vessel and *δ*_*e*_ is the vessel’s thickness. For short cylindrical blood vessels, the Pamm formula, commonly used in engineering, was utilized to calculate the critical value of the external pressure for vascular instability. This formula is as follows [43]:

9 $$P_{\text{cr}} = \frac{{2.59E\delta_{e}^{2} }}{{LD\sqrt {\frac{D}{{\delta_{e} }}} }}$$

where *E* is the Young’s modulus and *L* is the vessel’s length. For long cylindrical blood vessels, the formula of critical pressure for vascular instability is as follows [43]:

10 $$P_{\text{cr}} = \frac{2E}{{1 - \mu^{2} }}\left( {\frac{{\delta_{e} }}{D}} \right)^{3}$$

where *μ* is Poisson’s ratio. Based on physiological parameters of the external iliac artery, femoral artery, popliteal artery and tibial artery in the lower body, as shown in Table 6, the critical pressure for vascular instability of each part can be calculated [44–46].

**Table 6:** Parameters and critical pressures for vascular instability of lower body arteries

Parameter	External iliac artery	Femoral artery	Popliteal artery	Tibial artery
Internal diameter	8.900	5.000	4.730	2.500
Thickness	1.230	1.000	0.800	0.500
Length	103.410	297.290	167.340	158.670
Young’s modulus	1.750	1.750	1.750	1.750
Poisson’s ratio	0.499	0.499	0.499	0.499
Critical length	28.010	13.081	13.457	6.540
Container type	Long cylinder	Long cylinder	Long cylinder	Short cylinder
Critical pressure for vascular instability	92.288	279.697	169.155	9.584

Unit: internal diameter, thickness, length and critical length: mm, Young’s modulus: MPa, pressure: mmHg

The above calculation method of critical pressure for vascular instability was only for blood vessels without internal blood pressure. However, in actual human blood vessels, a pulsating blood pressure changes with time. When counterpulsation is applied, the pressure value required for vascular collapse should be the sum of the critical pressure for vascular instability and internal blood pressure at the current time point. Inflation time points for the cuffs wrapped around calves, thighs and buttocks were 0.25, 0.30 and 0.35 s, respectively, while the blood pressure for each part was 77.61, 78.32 and 108.38 mmHg, respectively. This means that the external pressure values required for collapse of the external iliac artery, femoral artery, popliteal artery and tibial artery were 200.668, 358.017, 246.765 and 87.194 mmHg, respectively. Due to differences in the personal physiological structure and indicators, these results may not be suitable for each patient. However, they could be used as a reference for the critical external pressure value of lower body vascular collapse for the majority of patients.

### Simulation of cerebral autoregulation

Cerebral autoregulation is an adaptive regulation function of cerebral blood vessels for blood pressure variation [5, 47, 48]. Due to the existence of cerebral autoregulation, there is no significant variation in CBF for healthy people when blood pressure is increased. However, in stroke patients, cerebral autoregulation is weaker than it is in healthy bodies. When counterpulsation is applied, the increased blood pressure will significantly increase the CBF during the diastole, effectively improving the cerebral ischemia condition. This is the treatment mechanism of EECP for stroke patients. The CBF formula is as follows:

11 $${\text{CBF}} = {\text{CPP}}/{\text{CVR}}$$

where CPP is cerebral perfusion pressure, and CVR is cerebral vascular resistance. The formula for CPP can be seen below:

12 $${\text{CPP}} = {\text{MAP}} - {\text{ICP}}$$

where MAP is mean arterial pressure, and ICP is intracranial pressure. The relationships between CBF, MAP and CVR can be deduced using the following formula:

13 $${\text{CBF}} = \left( {{\text{MAP}} - {\text{ICP}}} \right)/{\text{CVR}}$$

When blood pressure changes, the variation of ICP is not appreciable [49]; therefore, the variation of CPP depends on MAP. This means that the change in CVR is the main cause of cerebral autoregulation which maintains the stability of CBF during blood pressure changes. The authors of one clinical experiment found that cerebrovascular blood vessel lumen diameter variations correspond to blood pressure regulation [50]. When MAP increased by 30 mmHg, the average lumen diameter of the carotid artery, the proximal middle cerebral artery as well as the vertebral artery all decreased by approximately 4%, while the lumen diameter of the anterior cerebral artery and the distal middle cerebral artery decreased by 29% and 21%, respectively [50]. This means that, during EECP, an increase in MAP leads to an increase in CPP and varying degrees of adaptive contraction in cerebral arteries, thus increasing vascular resistance and maintaining CBF stability. The anterior cerebral and distal middle cerebral arteries contract much more than the vertebral and basilar arteries. Consequently, in the model, the resistances of the anterior cerebral (*R1_c* and *R1_b*) and distal middle cerebral arteries (*R2_d* and *R2_a*) increased significantly, while resistances of the internal carotid (*RA17* and *RA18*), proximal middle cerebral (*R1_d* and *R1_a*), vertebral (*RA19* and *RA20*) and posterior cerebral arteries (*R1_e* and *R1_f*) only showed a slight increase.

**Fig. 14:**
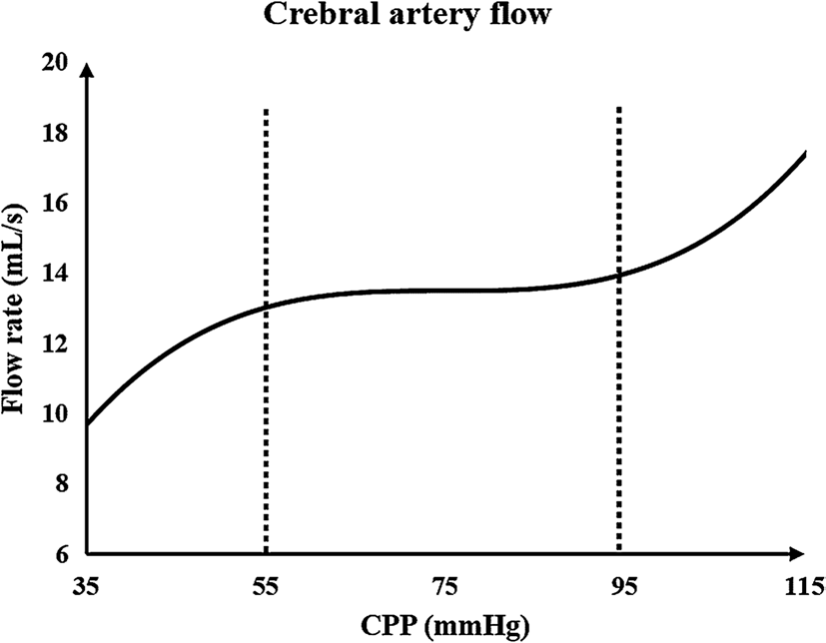
Typical diagram of the relationship between CPP and CBF. CPP is the cerebral perfusion pressure and CBF is cerebral blood flow

This qualitatively demonstrates that the resistance of each cerebral artery branch increases with the pulsation variation of blood pressure during counterpulsation. The quantitative variation in the resistance of each branch needs to be provided in the model. According to a typical diagram of the relationship between CPP and CBF [51], as shown in Fig. 14, when CPP was greater than 55 mmHg and less than 95 mmHg, CBF remained stable. It can therefore be assumed that cerebral vascular resistance increased linearly with increasing CPP within this range. When CPP was greater than 95 mmHg, CBF demonstrated a strong increasing trend, indicating that cerebral vascular resistance was stable at the threshold with a slight increase. According to this hypothesis, the equation for cerebral vascular resistance variation with CPP during counterpulsation is as follows:

14 $$R = \left\{ {\begin{array}{*{20}c} {k*\left( {{\text{CPP}} - 55} \right) ,\quad 55 \le {\text{CPP}} \le 95 } \\ {k*40 , \quad{\text{CPP}} > 95 } \\ \end{array} } \right.$$

where *R* is the variable resistance of each cerebral vascular branch and the coefficient *k* reflects the ability of cerebral autoregulation. Considering that the regulation abilities of patients with cerebral ischemic stroke are weaker than that of healthy individuals [47], *k* was set to 0.5 in the current study. By adjusting the variable resistance of each branch in the cerebral vascular microcirculatory structure in Fig. 10, variations of the cerebral vascular resistance along with blood pressure during counterpulsation could be simulated. The ICP waveform [52] was applied to voltage source Pe of the cerebral vascular microcirculation as shown in Fig. 10 and was used to simulate the intracranial pressure. By simulating the autoregulation mechanism, calculated waveforms before and during EECP were compared with clinical waveforms from our previous study [29]. The small difference confirmed the effectiveness of this method.

## Data Availability

The data were available.

## Abbreviations

EECP: enhanced external counterpulsation
0D: zero-dimensional
3D: three-dimensional
MAP: mean arterial pressure
CBF: cerebral blood flow
WSS: wall shear stress
FDA: Food and Drug Administration
SBP: systolic blood pressure
DBP: diastolic blood pressure
VECs: vascular endothelial cells
OSI: oscillatory shear index
WSSG: wall shear stress gradient
CTA: computed tomography angiography
CLV: capacitance of left ventricle
CRV: capacitance of right ventricle
CPP: cerebral perfusion pressure
CVR: cerebral vascular resistance
ICP: intracranial pressure
TAWSS: time-averaged WSS
TCD: transcranial Doppler
